# Lipidic Matrixes Containing Clove Essential Oil: Biological Activity, Microstructural and Textural Studies

**DOI:** 10.3390/molecules26092425

**Published:** 2021-04-22

**Authors:** John Rojas, Sergio Cabrera, Julie Benavides, Yasmín Lopera, Cristhian J. Yarce

**Affiliations:** 1 College of Pharmaceutical and Food Sciences, University of Antioquia, University Campus, Calle 67 No. 53-108, Medellín 050010, Colombia; sergiocabrera@gmail.com; 2 AOXLAB S.A.S, Cl. 32F #74b-122, Medellín 050030, Colombia; investigacion@aoxlab.com (J.B.); gerencia@aoxlab.com (Y.L.); 3 Laboratorio de Diseño y Formulación de Productos Químicos y Derivados, Departamento de Ciencias Farmacéuticas, Facultad de Ciencias Naturales, Universidad ICESI, Calle 18 No. 122-135, Cali 760035, Colombia; cjyarce@icesi.edu.co

**Keywords:** antioxidant and antimicrobial properties, clove essential oil, mechanical and wetting properties, cosmetic lipidic matrixes

## Abstract

Clove essential oil (CEO) is known for having excellent antioxidant and antimicrobial properties, but the poor stability of its components to light and temperature compromise this activity. The aim of this study is to evaluate the textural, antioxidant, antimicrobial and microstructural properties of matrixes produced with representative natural waxes and CEO. Thus, waxy emulsifiers, such as beeswax, candelilla wax, carnauba wax, and ozokerite wax, were employed to create such matrixes. The thermal, microstructural, textural, wetting, antioxidant, antimicrobial and infrared characteristics of the matrixes were then studied. The diverse chemical composition (long-chain wax esters in carnauba wax and short-chain fatty acids and hydrocarbons in beeswax and ozokerite wax, respectively) explained the differences in wetting, texture, melting, and crystallization characteristics. Crystal forms of these matrix systems varied from grainy, oval, to needle-like shape, but keeping an orthorhombic allomorph. The alignment and reorganization of beeswax and ozokerite wax into needle-like crystals increased the matrix strength and adhesion force compared to those of carnauba and candelilla matrixes, which showed weak strength and grainy morphology. The former two waxes and their matrixes also showed the largest plasticity. These lipidic matrixes show potential use for topical applications having acceptable antioxidant and textural properties.

## 1. Introduction

Essential oils (EOs) are natural, oily, colorless, and volatile liquids, which are soluble in alcohol, ether, and vegetable oils, but insoluble in water. These are found in the oil glands present at different depths in the leaves, flowers, buds, twigs, bark, fruits, seeds, and roots of numerous plants. These oils are commonly used in personal hygiene products and cosmetics. EOs are obtained by hydrodistillation, organic solvent extraction, cold pressing, and supercritical CO_2_ extraction from specific plant organs [1]. The extraction method, along with the origin, genotype, soil type, cultivar, climate, plant age, organ, or vegetative stage affects the quality and quantity of their components [2]. EOs are composed of complex mixtures of lipophilic compounds at different levels in which two or three major components usually prevail as compared to trace amounts of other compounds. Usually, the main compounds are responsible for the attributed biological properties [3]. Most commercial EOs are mainly chemotyped by gas chromatography (GC) and mass spectrometry (MS) analyses. EOs can also penetrate the skin and cause local effects, such as skin hydration and improved elasticity, possibly due to their accumulation in the fatty tissue after dermal application [4].

*Syzygium* is a genus of flowering plants that belongs to the myrtle family. The most commercialized *Syzygium* species are *Syzygium cumini* and *Syzygium aromaticum*. The oil content of *Syzygium* buds ranges between 10 and 20% (*w*/*v*) and it is mainly composed of a mixture of eugenol (72–92%), eugenol acetate (8–21%) and β-caryophyllene (2.8–8.6%) [5]. *Syzygium* oils possess antioxidant activity that prevents the damaging effect of air pollutants and UV radiation, including phototoxicity, skin aging, inflammation, and melanoma. Therefore, they could serve as substitutes of synthetic antioxidants, such as butylated hydroxyanisole (BHA), butylated hydroxytoluene (BHT), nitrites, and benzoates, which have shown harmful effects on human health. *Syzygium* oils help maintain the soft, youthful texture of skin, prevents the attack of harmful bacteria on the skin and improves the exfoliation of dead skin cells [6] In this scenario, clove essential oil (CEO) has been used in some preparations due to the antioxidant, anti-inflammatory, antimicrobial (*E. coli*, *Salmonella* spp., *Listeria monocytes*, *Campylobacter jejuni*, and *Neisseria gonorrhoeae*), antifungal (*Penicillium* spp., *Aspergillus* spp., *Trichophyton* spp., *Microsporum* spp., *Fusarium* spp., *Mucor* spp., *Candida albicans*, and *Epidermophyton floccosum*), and antiviral activities (herpes simplex, influenza A and hepatitis C viruses), to treat burns, wounds, pain and as a stimulant for the nerves [7]. CEO also increases lipid peroxidation and blood sugar levels in diabetic rats and reduces the tissue damage in the liver, and cardiac muscle of rats. The oil provides lubrication, and dispersion properties due to its high polarity [8].

Although this oil is considered as safe its components are highly volatile, water insoluble, and unstable to heat, oxygen, and light making the development of topical preparations challenging. Therefore, several strategies involving microparticulate systems have been developed to devise oily bioactive substances (OBS) using polymeric and lipidic materials. The former involves techniques, such as solvent evaporation, emulsion polymerization, and inclusion polymers using toxic solvents that yield impurities in the resulting product. Alternatively, lipidic vesicles, such as liposomes, ethosomes, and O/W emulsions, containing waxy compounds pose an innate instability since, in aqueous media, phospholipids and triglycerides can slowly become oxidized or hydrolyzed [9].

One feasible solution is the entrapment of the OBS into a colloidal lipidic matrix (LM) due to the solid-like state of these systems, reducing oil mobility and its leakage. LM protects the oil from degradation by skin enzymes and, thus, serves as a localized OBS delivery in some types of skin ailments [10]. Hot homogenization is the most widely used preparation method for these matrixes, due to the good reproducibility, high concentration of lipid phase, and absence of organic solvents. This method implies heating the oil and wax mixture above the lipid-melting point under homogenization or ultrasonication, followed by rapid cooling to achieve partial crystallization [11]. Thus, the incorporation of complex lipids, such as natural waxes into LM, enhances crystal formation, emulsification and improves the loading capacity compared to pure triglycerides. These waxes can form an isotropic LM with oils, cosolvents, and surfactants due to low polarity, long chain length, and high melting point of their compounds (Table 1) [12]. LM is stably resisting oxidation and hydrolysis, and contributes to the excellent skin tolerance, lubrication, and cleansing performance [13]. Waxes create a crystalline network within the LM that generates a framework with a specific hardness so that the LM does not break during application. LM is sticky and retains the oil because their endothermic events are close to the temperature of the skin, thus enabling the LM to spread during application. Waxes also contain essential fatty acids that contribute to the nourishment of dry skin. Further, they provide a barrier function mimicking the lamellar structure of the stratum corneum [14].

LM forms a film upon application on the skin, resulting in an occlusive effect, which in turn is proportional to the degree of crystallinity of the matrix. Thus, a high crystallinity and, hence, occlusive effect prevents OBS from evaporation allowing for deeper penetration into the skin. Moisturization occurs by inhibition of transepidermal water loss through occlusion [15]. Further, highly crystalline LM could act as physical sunscreens scattering or reflecting UV radiation [16]. LM can retain OBS without the need of additives due to the formation of intramolecular (H-bonding and polar-polar interactions) and intermolecular interactions (dipole-dipole interactions) between the constituents stabilizing the structure of the crystalline network [17]. CEO also functions as a penetration enhancer in transdermal bioactive delivery systems such as ibuprofen [18]. A moisturizer can form a hydrogen bond with a free water molecule. This property combined with the occlusive ability of waxes should create a LM with more lasting moisture protecting from drying. Using oil alone on the skin may cause irritation because of the oily nature, especially in people having an oily skin [19]. Therefore, a texturized LM offers great potential as medicated lip balm to moisturize and relieve chapped or dry lips, angular cheilitis, and cold sores.

This study evaluated the wetting, textural, antioxidant, antimicrobial and microstructural properties of LM produced with representative natural waxes and CEO. The topical application of these to matrixes allows for localized action, reduce the systemic risk of toxic effects, and increase patient compliance.

## 2. Materials and Methods

### 2.1. Materials

Steam-distilled clove essential oil was purchased from Smart Chemical (lot B-68623, Naucalpan, Mexico). Natural waxes were obtained from L&F (Medellín, Columbia). Sodium carbonate (lot J2180), potassium iodide (lot I0230) and dimethyl sulfoxide (lot DY938) were obtained from Honeywell (Charlotte, NC, USA). Sodium acetate trihydrate (lot 905067016), 2,4,6-tri-(2-pyridyl)-1,3,5-triazine (lot BCBW9015), 2,2-diphenyl-1-picrylhydrazil (lot 3430603), absolute ethanol (lot I1105110), acetic acid (lot K52005663), chloroform (lot K52039745), and phosphate buffer (lot AM1216473) were obtained from Merck (Darmstadt, Germany). Methanol (lot I1110418), Folin-Ciocalteu phenol reagent (lot LM02051903), and starch solution (lot GM01791711) were purchased from Loba Chemie (Mumbai, India). Further, (±)-6-hydroxy-2,5,7,8-tetramethylchromane-2-carboxylic acid (Trolox^®^) (97%, lot BCBQ2660V), gallic acid (lot SLBF8212V), tetrahydrofuran (lot 0001336590), diammonium salt (lot SLBH8520V), 2,2′-azobis (2-amidino-propane) dihydrochloride (lot MKBX9291V) and sodium fluorescein (lot MKBR1855V), were obtained from Sigma–Aldrich (St. Louis, MO, USA). Brain heart infusion broth (lot 17200206 BHI) was purchased from Micromedia LLC (Sofia, Bulgaria). Gentamicin (lot A02103C) was obtained from Genfar Laboratories (Cali, Columbia).

### 2.2. Preparation of Lipidic Matrixes

Approximately, a 15% wt wax dispersion was prepared by adding 45-mg wax to a CEO (~3 mL) solution. The samples were then heated at 80 °C under mild agitation (200 rpm) while using a heating plate coupled with a magnetic stirrer (Gehaka, MS7-H550-S, São Paulo, Brazil) until clear solutions were obtained (5 min). These colloidal dispersions were then allowed to cool down to 25 °C in a water bath within two minutes before testing.

### 2.3. FT-IR Characterization

Samples were characterized by Fourier transform infrared spectroscopy (FT-IR). A Nicolet FT-IR spectrometer (Thermo Fisher Scientific, Madison, WI, USA) was employed coupled with the Omnic^®^ v6.1 software (Thermo Nicolet Corporation, Waltham, MA, USA). Spectral data were collected at room temperature and accumulated from 16 scans with a resolution of 4 cm^−1^ in the range of 400 to 4000 cm^−1^. A correction was applied by subtracting the background spectrum of air. KBr pellets were prepared by grinding with ~5-mg sample on an agate mortar followed by compression at ~10,000 psi for 2 min.

### 2.4. X-ray Diffraction

Polymorphism was investigated on a PANalytical diffractometer (Empyrean 2012, Westborough, MA, USA) operated at 40 kV, and 30 mA, equipped with a monochromatic Cu Kα_1_ = 1.5460 A°, α_2_ = 1.54438 A° X-ray diffraction. Diffractograms were obtained over a 5°–35° 2Θ range and step scan and step time of 0.039 and 32 s, respectively. The degree of crystallinity was calculated while using the Peak fit software (Seasolve^®^ Inc., Framingham, MA, USA) by separating the crystalline and amorphous scattering radiation using the baseline selection tool.

### 2.5. Surface Properties and Wetting Behavior

Contact angle measurements were made on samples poured when melted into a 6 cm diameter Petri dish plates as described under Section 2.2. Contact angles were determined by the sessile drop method on a goniometer (OCA15EC, Dataphysics Instruments, Filderstadt, Germany) equipped with a driver software (vs. 4.5.14 SCA20, Dataphysics Instruments). Data capture was recorded on an IDS video camera (Imaging Development Systems GmbH, Obersulm, Germany), where a frame from 400 and 800 was taken as a reference point for measurement. Data capture was taken at 1 s and drop volumes ranged from 5 to 15 µL. Each measurement was conducted at 22 ± 1 °C and 60 ± 5% relative humidity. The test was conducted in triplicate. Isopropanol, ethylene glycol and ultrapure water was used to find the contact angles and they were then fitted to the Owens-Wendt-Rabel-Käelbe (OWRK) model. This model distinguishes between the surface free energy (SFE) in terms of nonpolar or dispersive attractive interactions (London interactions) and attractive interactions of polar type (dipole-dipole interactions or hydrogen bonding) [27]. It is expressed as:

(1) $$\frac{\gamma_{LV}\left(cos\theta \;+1\right)}{2{\left(\gamma_{LV}^{d}\right)}^{0.5}}={\left(\gamma_{SV}^{p}\right)}^{0.5}\frac{{\left(\gamma_{LV}^{p}\right)}^{0.5}}{2{\left(\gamma_{LV}^{d}\right)}^{0.5}}+{\left(\gamma_{SV}^{d}\right)}^{0.5}$$

where, the “*Y*” axis, “*X*” axis, slope and intercept correspond to: $y=\frac{\gamma_{LV}\left(cos\theta \;+1\right)}{2{\left(\gamma_{LV}^{d}\right)}^{0.5}}$,$\;x=\frac{{\left(\gamma_{LV}^{p}\right)}^{0.5}}{2{\left(\gamma_{LV}^{d}\right)}^{0.5}}$, $m={\left(\gamma_{SV}^{p}\right)}^{0.5}$, $b={\left(\gamma_{SV}^{d}\right)}^{0.5}$, respectively. The *p* and *d* superscripts correspond to the contributions of dispersive and polar interactions, respectively, which are known for each solvent.

### 2.6. Thermal Behavior

The thermal profiles of these matrix systems were examined with a DSC instrument (200PC, Nietzsche, Feinmahltechnik GmbH, Selb, Germany) equipped with a refrigerated cooling system. Nitrogen was used as the purge gas. The cell constant and temperature was set with indium. The sample (weighing from 9–12 mg/cup) was placed inside an aluminum pan and sealed with an aluminum lid. The samples were equilibrated at 5 °C and then heated to 80 °C (heating step) at a rate of 10 °C/minute followed by a cooling step to 5 °C at a rate of 10 °C/min. Characteristic parameters of the thermal curves, including the onset temperature (Tc_onset_ and Tm_onset_), peak maximum temperature (Tc and Tm), offset temperature (Tc_offset_ and Tm_offset_), melting and crystallization enthalpies (ΔHm and ΔHc), and ∆T (Tc_onset_-Tm_offset_) were obtained while using the Nietzsche Analysis software.

### 2.7. Phase Contrast Microscopy

The microstructure of these matrix systems was observed using an inverted phase contrast microscope (IN300, Amscope, Irvine, CA, USA) that was equipped with a color camera CCD-MT (Amscope Vs. 3.7, Corp, Irvine, CA, USA) at a 200× magnification.

### 2.8. Total Phenolic Content

These were determined while using the Folin–Ciocalteau method of the AOAC 2017.13 [28]. 100 µL of Folin–Ciocalteau reagent, 300 µL of 20% sodium carbonate solution, 15 mL of DI-water, and 50 µL of sample (0.1-g sample suspended in 100.0-mL methanol) was added and mixed. The mixture was incubated at 25 °C for 2 h and stored in the darkness. The resulting absorbance was measured at 765 nm in a spectrofluorophotometer (Synergy H1, Biotek^®^, Winooski, VT, USA). Methanol was used as negative controls. The total phenolic content in each formulation was determined using a standard curve prepared with gallic acid (40–200 mg/L) and expressed as milligrams of gallic acid equivalents (GAE) per 100-g powder.

### 2.9. Antioxidant Activity by 1,1-Diphenyl-2-picrylhydrazyl Method (DDPH)

The method of Hatano and Plank was applied with minor modifications [29]. Approximately, 0.5 mM solution of DPPH in methanol was prepared. Subsequently, 1 mL of this solution was added to 10-mL sample in methanol at a concentration of 0.1 mg/mL. The reaction mixture was shaken vigorously and allowed to stand in the dark at room temperature for 30 min followed by absorbance measurement at 517 nm using an UV/VIS spectrophotometer (UV-2602, Labomed, Inc., Culver City, CA, USA). Ascorbic acid was used as the reference standard. The standard curve was linear between 0.1 and 1.0 mM. The excipients dissolved in methanol were used as controls. The antioxidant effect was calculated as milligrams Trolox equivalent per 100 g of sample.

### 2.10. Antioxidant Activity by the Oxygen Radical Absorbance Capacity (ORAC)

It was conducted following the method described by the AOAC 2012.23 [30]. Approximately, 203.4 mg of 2,2′-azobis(2-amidinopropane) dihydrochloride (AAPH) was dissolved in 5-mL phosphate buffered to a final concentration of 75 mM, pH 7.4. A fluorescein stock solution (100 mM) was made in methanol and stored wrapped in foil at 4 °C. Immediately before use, the stock solution was diluted 1:200 with phosphate buffered and 150 μL of this sodium fluorescein solution was added to all sample wells. Further, blank wells received 25-μL phosphate buffered, whereas standard received 25 μL of Trolox^®^ (6-hydroxy-2,5,7,8-tetramethyl-chroman-2-carboxylic acid) solution. The mixtures were then placed in the florescence cuvette and preincubated at 37 °C for 30 min. Subsequently, 25 μL of AAPH solution was added to all wells (200 µL of final volume) and cuvettes were immediately put in the Biotek^®^ Synergy H1 fluorescence spectrophotometer. Fluorescence (excitation wavelength 485 nm, emission wavelength 530 nm) was recorded at 1-min intervals for 35 min. ORAC values were calculated as:

ORAC value = M_Trolox_/M_Sample_ × [(AUC_sample_ − AUC_blank_)/(AUC_Trolox_ − AUC_blank_)] (2)

where, M_Trolox_ is the molarity of Trolox. Final results were expressed in μM of Trolox equivalent per gram of sample (μmolTE/100 g).

### 2.11. Peroxide Value

The titrimetric method described in the AOAC 965.33 was employed [31]. Briefly, 5-g sample was dissolved in 30 mL of a mixture of acetic acid/chloroform (1:1) followed by the addition of 0.5 mL of saturated potassium iodide solution. The resulting mixture was stirred for 1 min and 30 mL of distilled water was added. Subsequently, this solution was titrated with 0.01 N sodium thiosulfate solution until almost all the yellow color disappeared. A 0.5 mL of 1% *w*/*v* starch solution was then added and titration was continued until complete disappearance of the blue coloration. Results were expressed as meq of oxygen/kg of sample.

### 2.12. Antimicrobial Activity

*Staphylococcus aureus* (ATCC 25923, Gram-positive) and *Escherichia coli* (ATCC 25922, Gram-negative) were chosen as representatives of the most common pathogenic bacteria involved in human topical infections. The broth microdilution methods, based on CLSI M07-A9 standard methodology were employed [32]. Briefly, 0.1-g sample was suspended in 10.0-mL DMSO. Samples were then diluted to obtain a final point well concentration from ~0.1 mg/mL to ~1 mg/mL. The inoculum was prepared by making a direct suspension in brain heart infusion broth (BHI) of a colony isolated from a 24 h nutrient agar culture. The broth was incubated at 37 °C for 24 h, followed by adjustment of optical density to 0.08 (0.5 on the McFarland scale, 1–2 × 10^8^ colony forming units (CFU/mL) using a spectrophotometer (Biotek^®^) at 625 nm. The percentage of inhibition was determined at 625 nm using a spectrophotometer coupled with a microplate reader (BioteK Synergy^®^) as follows:

Inhibition (%) = 100 × (A_ctrl_ − A_sample_)/A_ctrl_ (3)

Negative (distilled water with BHI broth) and positive control (bacterial inoculum and water) wells were also prepared. Gentamicin was used as a positive control to a final concentration of 40 mg/mL. The plaques were incubated at 37 °C for 24 h in normal atmosphere. The MIC was interpreted as the last well of each row where no visible bacterial growth was noticed (bacterial growth inhibition).

### 2.13. Mechanical Properties

Textural experiments were performed using a texture analyzer (CT3, Brookfield, Toronto, ON, Canada) with a penetration TA-P cylindrical probe. All samples were placed into the sample holders and the probe was positioned over the center of the cylindrical samples (35 mm × 9 mm), placed horizontally and moved down mm at 1 mm/s. Samples were evaluated at room temperature (25 °C). Wax and oil were mixed and heated to melt as described under “preparation of lipidic matrixes” and then poured into a tin mold. The cooled and solidified samples were stored at room temperature for 24 h before use. The analysis was conducted using the TexturePro CT^®^ software (Brookfield Engineering Labs, Inc., Middleboro, MA, USA). The stiffness was calculated from the slope of the linear region of the load-deflection curves, whereas the strain energy and toughness were found from the AUC of the linear region and the total region, respectively. The strength was found from the highest point from the curve.

### 2.14. Statistical Analysis

The analysis of variance was performed using the Minitab^®^ vs. 16 software (Minitab Inc., State College, PA, USA). A post-hoc analysis was then conducted to determine differences among treatment means using the Tuckey test (*p* < 0.05).

## 3. Results and Discussion

### 3.1. Crystallization Behavior

**Figure 1:**
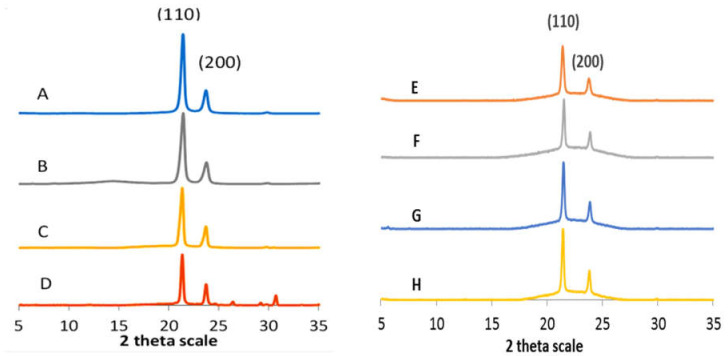
Wide angle X-ray diffraction for waxy emulsifiers. (**A**); carnauba wax (**B**); candelilla wax (**C**); beeswax (**D**) ozokerite wax (**E**); CRW matrix (**F**); CLW matrix (**G**); BEW matrix (**H**); OKW matrix.

Figure 1 depicts the wide angle X-ray diffractograms of neat waxes and CEO-based lipidic matrixes. Most waxes showed a degree of crystallinity (DC) ranging from 54 to 71%, whereas that of LM ranged from 21 to 32%. The latter is attributed to the poor crystal network ordering of the solid phase, due to the diverse presence of the oil components. Thus, LM had a weaker and less stable crystal structure. Carnauba wax (CRW) has virtually no hydrocarbon compounds prevailing wax ester (WEs) compounds, rendering a more ordered system explaining its large DC and melting temperature (82 °C). All the waxes and their LM exhibited similar wide angle diffraction patterns with one strong reflection (110) peak at 0.42 nm, followed by intensity by the second peak at 0.38 nm (200). The (110) reflection peak corresponds to a triple chain length stacking (3L), while the other peak (200) indicates a 2L packing. These results indicate a β orthorhombic crystalline sub-cell arrangement that is the most stable allomorph [21].

The contact angle is a parameter used to study the spreading phenomenon of a liquid on a solid surface. This phenomenon is expressed in terms of surface and interphase tensions of the solid-vapor and liquid-vapor surface tension. The contact angle is known as the angle formed by a drop of a reference liquid and the solid surface in the vapor-solid-liquid interphase [33]. Thus, wetting corresponds to the spreading phenomenon of a liquid on a solid surface. The wetting phenomena determine the interaction degree between the LM and skin. The Surface free energy (SFE) is obtained indirectly from the liquid-vapor surface values. Thus, a direct relationship between the contact angle and surface tension can be established using several semi-empirical models such as that proposed by Owens-Wendt-Rabel-Käelbe (OWRK) [34]. This model represents a balance between the cohesive and adhesive interactions occurring between the solid surface and the reference liquid. Thus, the balance between these two interactions renders different degrees of SFE.

Results of the SFE are shown in Table 2. Each analysis was conducted in terms of polar (attractive) or repulsive (hydrophobic) forces. In the case of pure waxes the magnitude of dispersive forces prevailed, except for beeswax, which showed balanced dispersive and polar forces. Interestingly, carnauba wax showed the largest total SFE values, due exclusively to the dispersive forces. The effect of an 85% CEO into the LM is reflected at increasing the magnitude of the polar forces, whereas the value of dispersive forces decreased. This effect was more drastic for CRW matrix, whereas the BEW matrix showed the largest increase in polar character. Interestingly, CRW and CLW matrixes exhibited the lowest and comparable degree of total SFE indicating an increase in the hydrophobic character, whereas BEW and OKW matrixes showed much larger values suggesting a more hydrophilic character having a better ability to interact via hydrogen bonding [35].

In the case of pure waxes, except for beeswax all waxes showed a hydrophobic character showing contact angles with water much larger than those from ethylene glycol and isopropanol. These results are explained in terms of the dielectric constant (ε) and the polar (*γ^P^*) and dispersive (*γ^d^*) surface tensions of reference liquids. For instance, ethylene glycol has an intermediate polarity (ε = 68) and closed to that of water resulting in a wetting pattern similar to that of water (ε = 80.1). Alternatively, isopropanol was the least polar solvent (ε = 17.9) rendering low contact angle values. As a result, the hydrophilic character of LM increased with the presence of CEO. This result is explained by the balance between *γ^P^* and *γ^d^*, where *γ^d^* was larger than *γ^P^* in polar solvents indicating a loss of hydrophobic repulsion and a gain of attractive dispersive interactions on the surface of the samples. This phenomenon was more prominent in isopropanol, which was the least polar solvent. 

Alternatively, the W_adh_ in water, which was the most polar solvent tested, increased as the formation of LM occurred. This phenomenon is due to the contribution of dispersive and polar forces as indicated by the polar/dispersive index (I_p/d_). This value indicates a balance between the polar and dispersive forces on the matrix surface. I_p/d_ values ≤ 1.0 indicate a prevalence for the London forces, whereas values larger than 1.0 indicate an incidence of polar interactions. Results show that for pure waxes beeswax and OKW had a mild polar character with I_p/d_ value close to ≥1, and this effect was more exacerbated in the respective LM due to the presence of CEO. Surprisingly, the incorporation on CEO into CLW was not sufficient to create a prevalent polar character into this LM.

**Figure 2:**
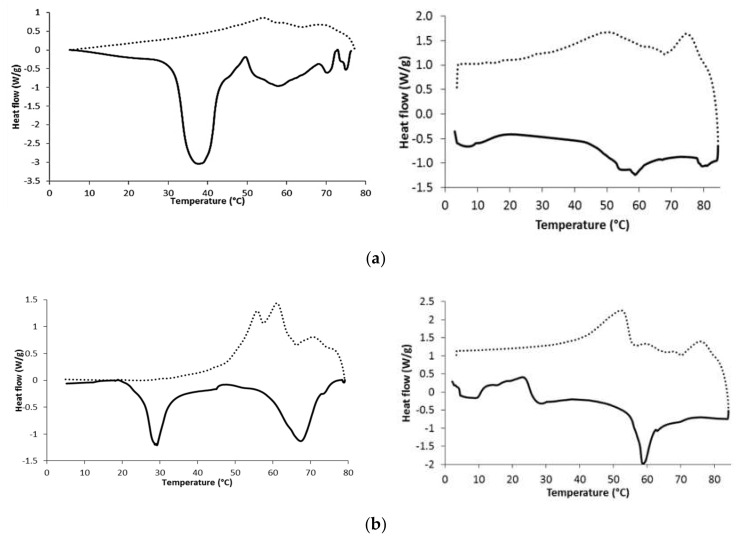

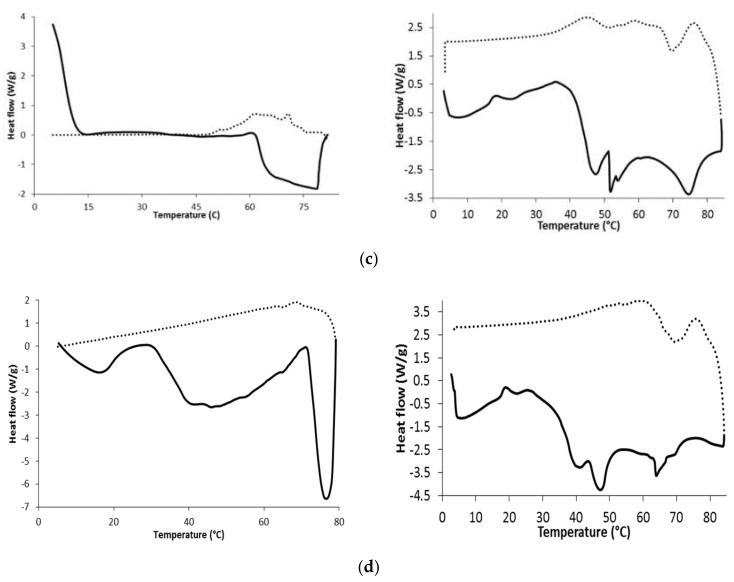
Differential scanning calorimetric profiles of neat waxes (left panel) and their lipidic matrix systems with lemon oil (right panel). Straight and dotted lines correspond to the heating and cooling modes, respectively. (**a**); beeswax (**b**); candelilla wax (**c**); carnauba wax, and (**d**); ozokerite wax.

Figure 2 depicts the thermograms that result from the crystallization profile (dotted lines) and melting profile (straight lines) of the neat waxes (left panel) and their matrix systems (right panel). The presence of different peaks is related to the heterogeneous chemical nature of the waxes. For instance, CRW is mainly composed of several types of WEs forming one large and wide melting event, whereas other materials, such as BEW and candelilla wax (CLW), also had a considerable number of low molecular weight HCs, FAs, and fatty alcohols (FAls), which resulted in melting events at lower temperatures. Thus, BEW showed a broad and intense melting peak at 37.8 °C due to the short-chain FAs, especially stearic acid C_18_, oleic acid C_18:1_, and palmitic acid C_16_, whereas at 57.9 °C showed a broad band attributed to the long chain WEs and HC fraction. Interestingly, neat OKW is deemed a mono-component wax, mainly comprised in 92% of HCs (Table 1), which explains the intense and narrow melting peak at a high temperature (76.6 °C) attributed to ceresin and resin fractions. Conversely, CLW exhibited two intense endothermic peaks at 29.3 and 67.4 °C, which could be due to melting of low molecular weight FAs and FAls fractions, and mid melting point compounds, such as HCs and WEs, respectively. Further, OKW showed a large fusion enthalpy (Table 3) that was attributed to the small steric interference of ceresin cyclic rings, petroleum resins, and alkane compounds favoring the formation of a compact structure.

Alternatively, the crystallization peaks of neat waxes were smaller in magnitude than the melting peaks. The presence of more than a single exothermic peak is also a result of the heterogeneous chemical composition. BEW, CRW, and OKW upon cooling crystallize, showing small bands without co-crystallization. Thus, crystallization in two stages is a consequence of the degree of crystalline disorder since, first, a group of compound crystals (mainly long chain WEs) is formed at a high temperature (68–71 °C), and then at a lower amount of energy the second arrangement by another set of compounds complete the crystal network. Such stepped crystallization results from hindered crystal growth, due to the low compatibility between major and minor components. The second and most important exothermic peak of BEW and CRW is shown at 53.9 and 61.5 °C, respectively, being ascribed to the crystallization of the FAc and FAl fractions, respectively [36].

Alternatively, CLW displayed multiple crystallization temperatures increasing the co-crystallization exothermic peak at 61.0 °C, followed by a small shoulder at 55.9 °C. During cooling, minor components, such as *n*-alkanes, nonacosane, and tritriacontane, form mixed molecular structures with the major alkane component, hentriacontane. This interaction affects molecular packing, which results in a broader co-crystallization peak. Thus, polar components such as FAls, FAs and WEs align and partially co-crystallized. This alignment is mainly driven by the formation of hydrogen bonds.

The right panel in Figure 2 shows the thermograms that resulted from the lipidic matrix systems. They are shown as more amorphous systems than neat waxes. In all cases, the multiple, broader and smaller endothermic events appeared suggesting amorphization. The HC, WEs, and FAl fraction of the CLW matrix eased crystallization at low temperatures. Matrixes having poor interaction with the terpenoids of CEO showed little crystallization due to poor molecular organization. In turn, it caused two small exothermic peaks. The first one initiated by the supersaturation of waxes in the matrix, followed by the second peak indicating complete crystallization. The magnitude of these two events depended on the wax chemical composition.

The energy that was required to crystallize and melt these matrix systems was smaller than that of the neat wax, which can be seen from enthalpy values. The melting enthalpy is a measurement of the amount of crystalline mass in these matrixes and depended on the wax chemical composition. OKW and the respective matrix exhibited the largest endothermic and exothermic enthalpies. The latter indicates a high resistance to temperature changes due to the high fraction of HC crystals formed.

The difference in temperature (∆T) between Tm_offset_-Tc_onset_, largely depended on the wax chemical composition. In neat waxes, CLW and CRW showed the largest ∆T, whereas BEW and OKW showed the lowest ∆T indicating a slow and rapid crystal formation, respectively. Further, in the OKW matrix the two crystallization events appeared before the melting events, suggesting a rapid network formation due to the large content of cyclic HCs. The low melting and crystallization enthalpies of the LM are due to the interaction of wax and CEO components. Thus, eugenol, which is the major component of CEO, has a large non-polar nature inhibiting the efficient ordering imparted by the lipidic waxes. Conversely, OKW compounds showed the largest non-affinity (hydrogen bond interactions) toward non-polar CEO molecules, resulting in a hindered solvatation. Thus, the single ring and polar moieties of eugenol try to intermingle with hydrocarbon molecules resulting in efficient ordering during the cooling process forming large crystalline networks. Alternatively, in CLW and CRW matrixes the polar region of eugenol eases the solubilization of WEs resulting in minimal crystal growth. This lesser interaction is due to the flat and more condensed cyclic core of eugenol, preventing, to some extent, interactions with the WEs and FAs, so they easily solvate while being incorporated into the matrix crystal structure in several stages. In fact, the waxy molecules should be partially insoluble with the oil to encourage crystallization, but, simultaneously, should be partially miscible within the oil to generate polar and hydrogen bond interactions [37].

### 3.2. FT-IR Characterization

**Figure 3:**
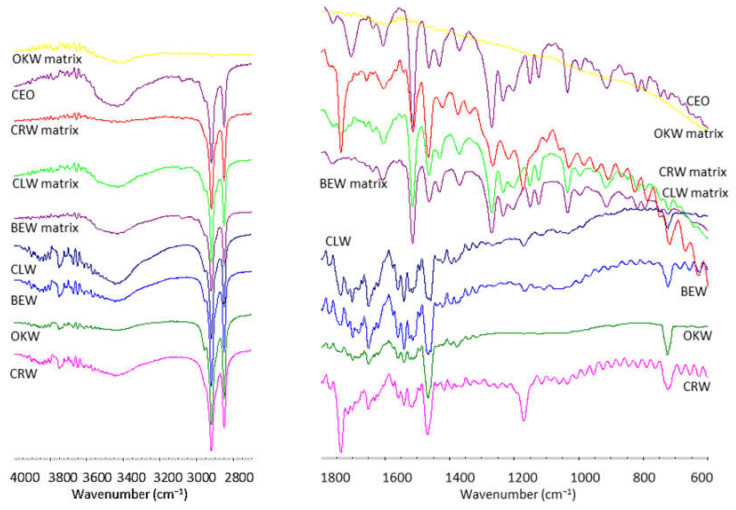
FT-IR spectra of neat waxes and clove essential oil matrixes prepared with beeswax, candelilla wax, carnauba wax, and ozokerite wax.

Figure 3 depicts the FT-IR spectra of waxes and their respective LM. The signature peaks of CEO are due to the main compounds such as eugenol, eugenol derivatives and β-caryophyllene (sesquiterpene). Thus, CEO exhibited bands at 3432, 3071, and 2920 cm^−1^ ascribed to O-H, =C-H, and C-H stretching, respectively. The peaks at 1637 and 1606 cm^−1^ originated from C=C stretching vibration of the aromatic moiety, whereas the peak at 1269 cm^−1^ specified to C-O stretching [38]. The spectral signal obtained at the frequencies of 1431, 990, and 804 cm^−1^ can be attributed to the presence of CH_2_ deformation vibration, CH out-of-plane, and ring deformation, respectively [39]. The stretching vibration absorption bands of the allyl group are located at 1637 and 995 cm^−1^. The band at 1765 cm^−1^ is assigned to the ester group C-O of eugenol acetate. The peak at 1027–1032 cm^−1^ is ascribed to CH-in-phase wag. The band between 1100 and 1210 cm^−1^ is assigned to the asymmetric C-O-C stretching of eugenol and its derivatives [40].

Most wax signals are attributed to their components such as esters, hydrocarbons, and free fatty acids. Thus, all neat waxes exhibited a broad band at 3445 cm^−1^ attributed to the intermolecular -OH group vibration. They also showed absorption bands at 2920 (CH_2_ asymmetric stretching), 2850 (CH_2_ symmetric stretching), 1465 (CH_2_ scissor deformation) and 724 cm^−1^ (CH_2_ rocking mode) corresponding to hydrocarbons, especially for ozokerite wax. These are attributed to ceresin, which is the major component of ozokerite wax [41]. The signals related to the ester and free fatty acids vibrations at 1738 (monoesters), 1702 (free fatty acids) and 1170 cm^−1^ (C=O stretching and CH bending) were present in beeswax, carnauba wax and candelilla wax, but absent in ozokerite wax [42]. Particularly, carnauba wax showed stronger absorption bands corresponding to ester vibrations at 1738 and 1170 cm^−1^. Further, neat waxes also showed the C=C vibration at 1651 cm^−1^ indicating some degree of unsaturation [43].

Alternatively, the FT-IR spectra of wax-based matrixes are complex since most CEO and waxes vibrational modes mixed and overlapped. Thus, the strong C=O stretching band of CEO at 1760 cm^−1^ disappeared, whereas the CH_2_ bands at 2850, 2920, and 3432 cm^−1^ remained intact. Further, some particular bands at 1513, 1464, and 1268 cm^−1^ shifted and increased in magnitude. Conversely, bands at 1604, 1430, 1152, 1034, and 996 cm^−1^ reduced in intensity. This finding is due to superposition of wax and CEO fingerprints. Similar results have been observed in wax-based systems such as nanoemulsions [44].

### 3.3. Crystal Morphology

**Figure 4:**
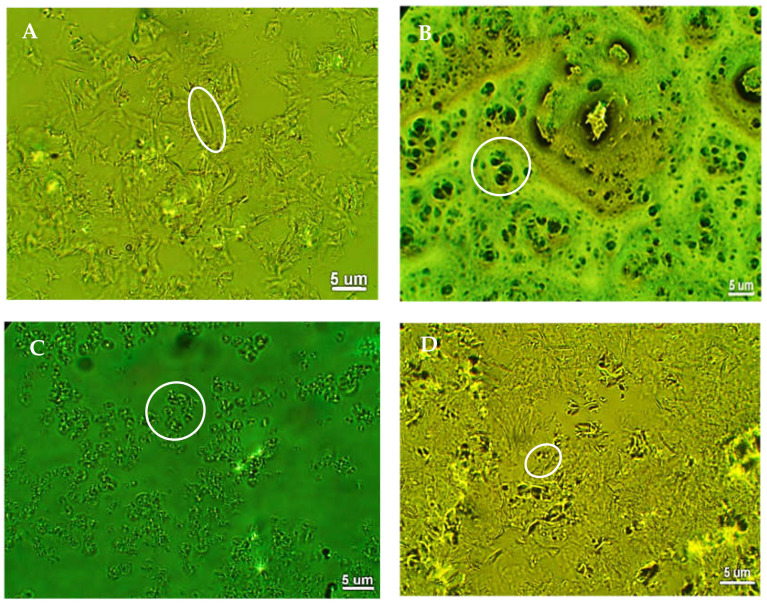
Phase contrast microphotographs of clove essential oil matrixes prepared with beeswax (**A**); candelilla wax (**B**); carnauba wax (**C**); and (**D**); ozokerite wax.

Figure 4 depicts the morphological features of the LM, as seen by the phase contrast microphotographs. The polarity of the emulsifying wax affected the magnitude of crystal formation and self-assembling since OKW and BEW wax having low polarity assembles into short and dispersed fibers that grow and become interconnected, whereas more polar waxes formed conglomerates due to free mobility in the medium. Thus, a clear balance between hydrogen bonds and hydrophobic interactions was critical easing self-assembling and the development of a defined microarchitecture. For instance, BEW matrix crystallized into a fine needle inbreeding together, which is further organized into an open aggregate-like structure. These needles were thin, elongated, and bent having 5–15 µm in length. This particular shape formed a honey comb or porous system that was responsible for the good consistency and moderate degree of crystallinity of this LM. Conversely, the OKW matrix was composed of stacked fibrous crystals of ~5–15 μm in size. Further, partial aggregates are also visible due to a two-stage crystallization process that was initiated by a three-dimensional nucleation center followed by the organization of needle-like crystals in the outer layer, resulting in striated growth.

Alternatively, CLW matrix mostly developed small oblong-like crystals rather than fibers of 2–10 μm in size. This morphology is the result of the HC components that co-crystallized with the WEs fraction during cooling. Likewise, CRW matrix had an anisotropic, grain-like morphology with length ranging from 2 to 10 µm. This material also exhibited a high content of WEs, making crystalline particles at the junction zones and within the network to reorganize into a loose molecular packing. In turn, this poor level of structural organization was responsible for the smallest degree of crystallization and poor strength of such matrixes. These results agreed with those previously published where morphology was rather dependent on the waxy emulsifier employed rather than the type of essential oil [45].

### 3.4. Antioxidant Properties

Polyphenol-rich products protect the skin when exposed to oxidative conditions of UV light. Such protection has been associated with the capacity of polyphenols to neutralize peroxyl radicals generated during lipid peroxidation. Antioxidant compounds such as phenolic acids, polyphenols, and flavonoids scavenge free radicals such as peroxide, hydroperoxide, or lipid peroxyl and thus inhibit the oxidative mechanisms that lead to degenerative diseases. Table 4 lists the antioxidant activity of waxy of LM. This activity was preserved in LM containing beeswax, whereas the OKW matrixes having the largest content of hydrocarbons showed low antioxidant activity, independent of the method employed. To explain the large difference of data between two antioxidant techniques, the fundamentals of each method should be taken into account.

The ORAC method measures the radical chain breaking capacity of LM by checking the inhibition of oxidation induced by peroxyl radical. These radicals are the main free radicals responsible for lipid oxidation in biological systems. In this test, the peroxyl radical produced by the initiator (AAPH, 2,2′-azobis(2-amidinopropane) dihydrochloride) reacts with a fluorescent molecule, resulting in the loss of fluorescence. Thus, AAPH undergoes thermal breakdown in the presence of oxygen to generate peroxyl radicals as oxidants that further react with fluorescein and LM. 

The area under the curves in the presence of antioxidants is compared to the area of a blank indicating the peroxyl radical scavenging capacity of the LM. Since LM has a lipophilic nature, methanol was needed to solubilize the lipophilic phenolic compounds. Some studies have reported the antioxidant capacity for CEO and eugenol of 2.4 g Trolox/g and 1.94 g Trolox/g, respectively. This result indicates that the total antioxidant activity of the polyphenol mixture is a reflex of the individual components activity [46]. Another study showed a value of 40,000 µmol TE/g for eugenol, which is close to the value obtained in this study indicating a high contribution of this molecule into the CEO [47].

Independent of the antioxidant method, BEW-based matrix showed the highest antioxidant activity and content of total phenolics, whereas OKW-based matrix presented the lowest activity and phenolics. However, there was no correlation between the content of phenolics and antioxidant activity for CEO. In fact, all LM had a larger content of phenolics than CEO indicating also a high reactivity of the wax components with the Folin–Ciocalteau reagent, especially for the BEW-based matrix. However, it is deduced that not all phenolic compounds present in waxes had a net antioxidant ability since CEO alone showed the largest antioxidant ability according to the DDPH assay. Researchers have reported a CEO phenolic content of 21,751 mg of tannic acid equivalents/100 g at 100 µg/mL concentration, which is higher than the one reported in this study [48].

The present study found large difference between the antioxidant values resulted from the ORAC and DDPH methods. In fact, the ORAC method showed numerically much higher values compared to the DPPH method, where the values were mathematically lower, indicating the greater sensitivity of the fluorescence ORAC method in detecting antioxidant activity. Further, the incubation period at 37 °C in the ORAC method also facilitated full extraction of most antioxidant compounds, including fat-soluble materials. Therefore, these two methods provide an independent, nonequivalent, antioxidant activity. It is feasible that each method renders a different profile depending on the chemical structures and the measurement conditions.

The principle of the DPPH method is the generation of a free radical causing a violet solution in methanol, which then reacts with antioxidant leading to chain breakage and as a result, color changes to light yellow (DDPH-H). Results were expressed compared with the vitamin E analog Trolox ((*S*)-(-)-6-hydroxy-2,5,7,8-tetramethylchroman- 2-carboxylic acid]. DDPH is highly stable and has a large redox potential being able to oxidize a large variety of natural antioxidants. Some authors have found CEO values of radical scavenging from 50–67% using the DDPH method that depended on the concentration (15–45 µg/mL) and plant-flowering stage [49], since other studies reported phenolic amount of 240.70 mg GAE/100 g in clove fruits and a free radical scavenging of 87.5% by DDPH. These values are lower than the values found for pure CEO in this study (7289.2 GAE/100 g and 12,002.2 mg TE/100 g) [50]. The radical scavenger potential of CEO is due to polar polyphenols such as eugenol and eugenol acetate. These compounds work synergistically with each other to produce a broad spectrum of antioxidant activities that create an effective defense system against free radical attack. Particularly, these compounds allow for the donation of a hydrogen atom and subsequent stabilization of the phenoxyl radical generated creating stable compounds that do not start or propagate oxidation. These two molecules possess some conjugation of the carbon chain with the aromatic ring that participates in the stabilization of the phenoxyl radical by resonance. Eugenol reduces two or more DPPH radicals, despite having only one hydrogen from a hydroxyl group. Further development of dehydrodieugenol (dimers) with two phenolic hydroxyl groups originating from eugenol intermediate radicals has also been proposed as mechanism between eugenol and DPPH radicals [51]. Compounds reactive with the hydroxyl showed that eugenol competes with these reactive compounds for this radical and thus, it stabilizes wax components such as fatty acids, and protects the skin from damage caused by UV-light [52].

Peroxides have damaging effects on people skin and contribute to different diseases cancers and allergies. The oxidation process occurs when there is a contact between oxygen in the air with vegetable oils, essential oil or fats. The peroxide value is a factor stating the content of oxygen as hydroperoxides in a substance and determines the resistance of samples to oxidation in the presence of an antioxidant. In fact, the peroxide value measures the oxidation present. The sample is treated with a mixture of acetic acid and chloroform and then with a solution of potassium iodide. The latter compound is oxidized by the compounds present in the sample and iodine is released and titrated with sodium thiosulfate. Essential oils should only be used when the level of hydroperoxide value is kept under 20 mmoles of active oxygen per liter, or less than 15 for fatty acids. Thus, LM resulted more stable than raw CEO showing a peroxide value of 13 meqO/kg, whereas LM showed no signs of oxidation, indicating an improvement of stability, even though waxes such as beeswax, carnauba and candelilla have some fraction of fatty acid components. CEO has been used to delay the oxidation of hazelnut oil stored at 50 °C in darkness showing values of <20 meqO/kg for 7 days [53]. The variations of antioxidant potential CEO is due to different polarities of volatile and nonvolatile compounds found within the CEO. Autoxidation also proceeds through an attack at the methylene group adjacent to the double bond leading to the formation of allylic hydroperoxides. The addition of oxygen occurs by a radical chain reaction on the carbon adjacent to the double bond and the radical is the result of the removal of hydrogen from the fatty acid. Further, unsaturated fatty acids oxidize to a greater extent than saturated fatty acids and methyl esters. Once oxidized, the decomposition of fatty acids occurs along with the conversion of hydroperoxides into aldehydes and ketones and free acids resulting in rancidity [54]. Oxidation also occurs by high temperatures, resulting in the formation of hydrogen peroxide, and carbonyl component such as aldehydes [55].

CEO had low antimicrobial activity against *E. coli* (56.2%) and *S. aureus* (52.9%) compared to synthetic wide spectrum antibiotic (gentamicin), which showed a 97% inhibition of bacterial growth. This result is ascribed to the ability of gentamicin to diffuse through the transporter channels of the bacterial membrane and interact with the 30 s ribosomal subunit disrupting the process of protein bacterial synthesis. Conversely, polyphenol compounds from CEO might not have an appropriate transporter to pass through the bacterial wall to induce their antimicrobial ability. A recent study conducted at 21 °C and 37 °C for CEO showed a large antimicrobial activity at 37 °C due to the large fluidity of the lipid layer of the bacteria, which in turn depended on the proportion of unsaturated fatty acids. As a result, CEO is permeable into the membrane and accumulates within destroying to the membrane [56]. Recent studies on CEO found 81% inhibition of both strains with respect to streptomycin, but better action against *C. albicans* (100%) with respect to amphotericin B (70.3%). They also found a MIC of 0.13% for all strains, whereas this study found a MIC of 0.16 ± 0.02% and 0.15 ± 0.02% for *E. coli* and *S. aureus* strains, respectively [57]. As mentioned previously, these minor variations in activity are due to factors such as the extraction method, origin, genotype, soil type, cultivar, climate, plant age, organ, or vegetative stage of the clove tree.

### 3.5. Mechanical Properties

**Figure 5:**
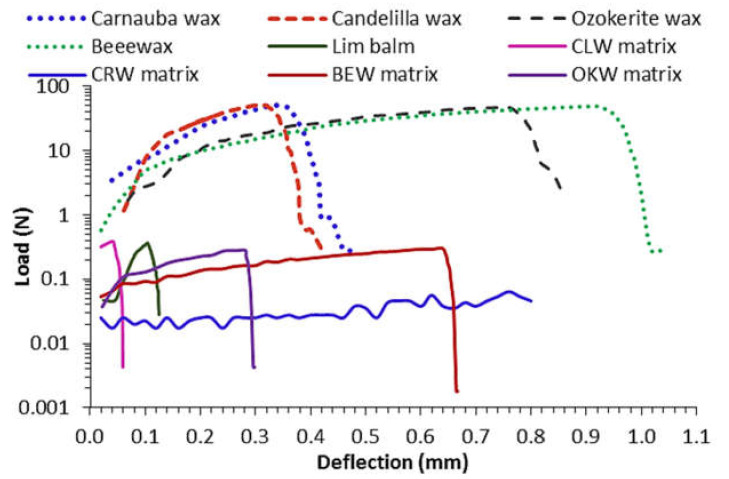
Load-deflection curves of lipidic matrixes made of clove essential oil matrixes with several waxy emulsifiers.

The mechanical properties were evaluated on cylindrical matrixes of ~2.3 g. The load-deflection curves are depicted in Figure 5. Ductile materials exhibit a linear stress-strain relationship up to a well-defined yield point. The linear upward portion of the curve is the elastic region where the deformation is recoverable. After the yield point, plastic deformation occurs, which is permanent and irreversible. As deformation continues, the stress increases because of strain hardening until it reaches the breaking strength. All cylindrical matrixes made of pure waxes showed no air holes, sweating, blooming, sagging or cracking. Conversely, clove-based matrixes were weaker in nature. Table 5 lists the results of the mechanical properties. Beeswax, ozokerite and their respective lipidic matrixes showed the largest toughness and elastic energy, which in turn, is translated in a good spreadability under application with modest pressure. Further carnauba and candelilla waxes showed the largest stiffness, but small elastic limits indicating a more brittle behavior. Interestingly, carnauba matrix showed the softest behavior showing a large ability to absorb energy and virtually no resistance to penetration. Here, weaker LM was obtained using those texturized with waxes containing a larger number of polar moieties (i.e., esters and alcohols) prevailing solubilization rather than crystallization. The crystallization of LM containing CRW was difficult to control as they had either a porous nature or significant shrinkage on the surface, or were brittle on the surface but smooth in the interior. BEW and OKW underwent plastic deformation once the stress exceeded its peak force due to a fibrous lamellar structure. In contrast, when the curves of CLW and CRW reached a peak force, a sharp decrease in force occurred, indicating that these grainy samples had a brittle character.

This singularity is explained by the high content of polar moieties such as esters and fatty alcohols. In art application, certain plasticity is required so that the LM can be manipulated to provide a good running or spreadability. The complicated molecular mixture in LM results in a unique crystalline and amorphous structure. The branching of the waxes long chains forms rigid semicrystalline zones, whereas the mobile amorphous zones contain liquid components of the CEO. 

The commercial product showed a clear stiffness with a rapid crack. Thus, it is considered a brittle material. Usually, if the LM is too stiff, it cannot be applied evenly. Conversely, when the LM is too runny, it does not stay long on the skin. The low endothermic events of CEO-based ingredients (55–65 °C) allows for the LM to lasts longer on the skin.

## 4. Conclusions

The diverse chemical composition of lipidic matrixes resulted in different crystallization profiles and improved clove essential oil stability by inhibiting the lipid peroxidation and scavenging of proton, oxy, and hydroxyl radicals. The physicochemical properties of these matrixes were modulated by the innate chemical composition of waxy materials. Thus, the predominant short-chain HC in BEW, and OKW resulted in the most crystalline and strongest materials, whereas the high number of long-chain WEs in CRW and CLW matrixes resulted in a less crystalline and weaker material. Waxy materials can be employed for the formulation of topical preparations, with this oil having a modulated texture, cleansing, and antioxidant properties.

## Figures and Tables

**Table 1:** Chemical composition of waxy emulsifiers.

Waxy Emulsifier	Aliphatic Ester (%)	Hydroxy Esters (%)	Diesters (%)	Hydrocarbon (%)	Fatty Alcohol (%)	Free Fatty Acids (%)	Ref.
Beeswax	C_40–0_: 35	C_24–34_: 12	C_54–68_: 17	C_21–35_: 14	C_24–36_: 1	C_16,_C_24,_C_34_: 14	[20,21]
Candelilla	Simple esters-lactones: 21	6–8	0	hentriacontane: 46–46.5	12–14	5	[22,23]
				nonacosane: 2.5			
				triacontane: 2.5			
Carnauba	C_50_: 38–40	13	* 4-HCA: 21 ** 4-MCA: 7	C_27–28_: 1	C_32–34_: 12	C_16_: 5	[24,25]
Ozokerite	0	0	0	asphaltens: 1–3	0	0	[26]
				cerecin: 40–60			
				mineral oil: 25–45			
				parafin: 1			
				petroleum resins: 12			

* *p*—hydroxycinnamic aliphatic di-esters, ** *p*—methoxycinnamic aliphatic diesters.

**Table 2:** Parameters obtained from the XRD profiles and surface free energy (SFE) measurements.

Wax Material or Lipidic Matrix	DC (%)	(110°)	*d*-Spacing (nm)	(200°)	*d*-Spacing (nm)	Total SFE (mN/m)	SFE^d^ (mN/m)	SFE^p^ (mN/m)	r^2^	I_p/d_	Wadh (mN/m)
BEW	53.7	21.35	0.417	23.71	0.376	* 20.4 ± 1.0	10.0 ± 0.0	10.4 ± 1.0	1.00	1.04	74.6 ± 1.5
OKW	67.7	21.35	0.417	23.71	0.376	* 20.5 ± 1.1	* 20.4 ± 1.1	* 0.05 ± 0.0	0.91	2.45	* 45.5 ± 5.1
CRW	70.6	21.40	0.416	23.71	0.376	116.5 ± 6.9	116.5 ± 6.9	* 0.0 ± 0.0	0.89	* 0.00	* 47.2 ± 15.3
CLW	67.1	21.4	0.416	23.77	0.375	* 18.8 ± 1.5	* 18.0± 1.4	* 0.8 ± 0.0	0.94	* 0.04	* 51.0 ± 1.2
BEW matrix	29.0	21.47	0.415	23.88	0.374	47.0 ± 4.1	5.7 ± 0.1	41.3 ± 4.0	0.96	7.24	113.3 ±5.5
CRW matrix	28.5	21.41	0.416	23.78	0.375	* 26.1 ± 1.2	3.5 ± 0.1	22.6 ± 1.1	0.95	6.45	85.1 ± 2.5
CLW matrix	20.6	21.52	0.414	23.88	0.374	* 23.9 ± 1.2	* 17.0 ± 1.0	6.9 ± 0.1	0.99	* 0.41	74.5 ± 8.7
OKWmatrix	31.6	21.47	0.415	23.83	0.374	31.4 ± 2.2	* 14.9 ± 1.1	16.4 ± 0.1	0.91	1.10	92.3 ± 2.8
*p-value*	N.A.	N.A.	N.A.	N.A.	N.A.	0.00	0.00	0.00	N.A.	0.00	0.00

*SFE^d^* and *SFE*^p^, correspond to dispersive and polar contribution in total SFE. r^2^ corresponds to the linear determination coefficient for SFE by the OWRK model. Liquid parameters: Water (*γ_total_ =* 72.1 mN/m, *γ^D^ =* 19.9 mN/m, *γ^P^ =* 52.2 mN/m and ε = 80.1); Ethyleneglycol (*γ_total_ =* 48.0 mN/m, *γ^D^ =* 29.0 mN/m, *γ^P^ =* 19.0 mN/m and ε = 68); and Isopropanol (*γ_total_ =* 23.0 mN/m, *γ^D^ =* 19.5 mN/m, *γ^P^ =* 3.5 mN/m and ε = 17.9). Wadh, work of adhesion in water. Values are expressed as mean ± standard deviation (SD) (*n* = 3). * values within each column are not statistically significant according to the Tuckey test.

**Table 3:** Thermal parameters obtained from the DSC profiles.

Wax Material or Lipidic Matrix	Tm _Onset_ (°C)	Tm (°C)	Tm _offset_ (°C)	Tc _onset_ (°C)	Tc (°C)	Tc _offset_ (°C)	∆T (°C)	ΔHm (J/g)	ΔHc (J/g)
BEW	31.4	37.8	43.8	57.7	53.9	48.5	13.9	26.0	0.8
CLW	25.7	29.3	33.2	57.0	59.9 *	45.4	23.8	5.6	1.3
	55.5	67.4	72.3	65.7	61.0	57.0	6.6	6.6	1.6
CRW	60.0	82	85.0	71.7	61.5	51.7	13.3	17.0	0.4
OKW	70.1	76.6	79.0	65.8	64.0	57.9	13.2	29.2	1.1
BEW matrix	4.0 51.9 77.0	7.1 57.9 74.6	8.4 60.7 82.8	- 61.5 79.5	- 50.7 74.6	- 35.2 70.9	- 0.8 2.5	0.9 3.0 0.34	- 2.8 0.9
CLW matrix	2.9 22.6 53.5	9.0 27.5 58.7	11.0 29.2 62.1	- 56.8 80.9	- 52.3 75.8	- 40.3 71.7	- 27.6 18.8	1.2 3.5 3.8	- 4.4 2.2
CRW matrix	3.1 40.4 50.3 65.5	7.2 47.5 51.8 74.6	12.3 50.7 57.9 79.1	- 52.3 64.0 81.6	- 45.0 58.6 77.5	- 38.4 55.3 76.1	- 1.6 6.1 2.5	0.5 2.4 5.0 7.3	- 1.6 0.4 1.7
OKW matrix	3.2 35.8 42.0 61.0	5.9 41.1 47.4 63.9	13.6 42.0 50.2 66.9	- - 67.1 81.2	- - 58.5 75.6	- - 55.3 71.8	- - 16.9 14.3	0.3 3.3 5.8 1.6	- - 2.1 2.3

* A shoulder from the main peak.

**Table 4:** Antioxidant properties of the wax-based matrixes.

Sample	Total Phenolic Content (mg Gallic Acid/100 g)	DDPH (mg Trolox/100 g)	ORAC (mg Trolox/100 g)	Peroxide Value (meq Oxygen/kg)
BEW matrix	480,165.6 ± 18.3	8504.1 ± 66.5	247,129.8 ± 0.9	0 ± 0
CRW matrix	427,453.2 ± 18.2	7209.9 ± 45.7	205,140.7 ± 0.8	0 ± 0
CLW matrix	383,993.4 ± 14.8	6765.1 ± 47.3	170,429.0 ± 0.0	0 ± 0
OKW matrix	370,787.4 ± 15.0	5143.8 ± 55.3	159,944.7 ± 1.0	0 ± 0
CEO	7289.9 ± 11.3	12,002.2 ± 44.3	N.R.*	13.02 ± 0.01
*p-value*	0.00	0.00	0.00	0.00

Values are expressed as mean ± standard deviation (SD) (*n* = 3). * Not reported since values were outside the range of the calibration curve.

**Table 5:** Mechanical properties of lipidic matrixes.

Wax Material or Lipidic Matrix	Mechanical Properties				
	Elastic Energy (J)	Stiffness (N/mm)	Ultimate Strength (N)	Elastic Limit (mm)	Toughness (J)
Beewax	22.3	57.6	48.7	0.9	25.4
Candelilla	6.1	196.0	50.3	0.3	8.5
Carnauba	6.0	203	50.4	0.32	9.0
Ozokerite	17.7	70.5	49.0	0.72	21.7
BEW matrix	0.11	0.40	0.30	0.62	0.12
CLW matrix	0.006	3.3	0.39	0.04	0.011
CRW matrix	0.022	0.04	0.06	0.74	0.025
OKW matrix	0.04	0.98	0.28	0.26	0.05
Commercial product	0.01	6.0	0.37	0.1	0.02

## Data Availability

The data presented in this study are available on request from the corresponding author.

## Sample Availability

Samples of the compounds are available from the authors.
